# Dietary Patterns and Household Food Insecurity in Rural Populations of Kilosa District, Tanzania

**DOI:** 10.1371/journal.pone.0126038

**Published:** 2015-05-21

**Authors:** Julius Edward Ntwenya, Joyce Kinabo, John Msuya, Peter Mamiro, Zahara Saidi Majili

**Affiliations:** 1 University of Dodoma, Department of Public Health, P.O. BOX 395, Dodoma. Tanzania; 2 Sokoine University of Agriculture, Department of Food Science and Technology P.O. Box 3006, Morogoro, Tanzania; TNO, NETHERLANDS

## Abstract

**Introduction:**

Few studies have investigated the relationship between dietary pattern and household food insecurity. The objective of the present analysis was to describe the food consumption patterns and to relate these with the prevalence of food insecurity in the context of a rural community.

**Methodology:**

Three hundred and seven (307) randomly selected households in Kilosa district participated in the study. Data were collected during the rainy season (February–May) and post harvest season (September–October) in the year 2011. Food consumption pattern was determined using a 24-h dietary recall method. Food insecurity data were based on the 30 day recall experience to food insecurity in the household. Factor analysis method using Principal Components extraction function was used to derive the dietary patterns and correlation analysis was used to establish the existing relationship between household food insecurity and dietary patterns factor score.

**Results:**

Four food consumption patterns namely (I) Meat and milk; (II) Pulses, legumes, nuts and cooking oils; (III) fish (and other sea foods), roots and tubers; (IV) Cereals, vegetables and fruits consumption patterns were identified during harvest season. Dietary patterns identified during the rainy season were as follows: (I) Fruits, cooking oils, fats, roots and tubers (II) Eggs, meat, milk and milk products (III) Fish, other sea foods, vegetables, roots and tubers and (IV) Pulses, legumes, nuts, cereals and vegetables. Household food insecurity was 80% and 69% during rainy and harvest–seasons, respectively (P = 0.01). Household food insecurity access scale score was negatively correlated with the factor scores on household dietary diversity.

**Conclusion:**

Food consumption patterns and food insecurity varied by seasons with worst scenarios most prevalent during the rainy season. The risk for inadequate dietary diversity was higher among food insecure households compared to food secure households. Effort geared at alleviating household food insecurity could contribute to consumption of a wide range of food items at the household level.

## Introduction

Assessment of dietary patterns is becoming popular in the field of nutrition. Dietary patterns are being increasingly examined as predictors of disease outcomes in various settings. Despite the recent popularity in the use of dietary patterns to investigate diet–disease associations, the relation between dietary patterns and food insecurity have not been fully explored. Dietary patterns facilitate the study of the whole diet, recognizing that people consume foods in combination [1]. Diet is further described as a multidimensional exposure and thus it is not appropriate to attribute differential disease prevalence or symptoms to a single nutrient or food group [2]. Thus the approach is contrary to the assessment of the individual food groups recognising that foods are consumed in combination. There is worldwide growing concern over the change in dietary pattern. The changes have been linked with nutrition transition in developing countries characterised with a co- existence of both over nutrition and under nutrition [3]. However, little is known on the current situation in most of rural settings and the way through which food consumption pattern are related to the level of food insecurity. The well recorded shift in dietary pattern that is associated with existence of the double burden of malnutrition call for a need to establish the existing linkages between dietary patterns and food insecurity. Limited understanding of the potential linkages between food insecurity and actual food consumption in most communities limits possibility for developing potential interventions that links agriculture and nutrition. Thus leaving the two fields not connected is likely to perpetuate the intellectual vacuum in terms of potential synergistic effect the two fields have in promoting human wellbeing.

Factor analysis method is commonly-used to derive dietary patterns by reducing data into small components [4]. Factor analysis utilizes correlations that exist between different food groups to derive principal components by identifying linear combinations of foods that are frequently consumed together. The components are therefore utilized to deduce dietary patterns. Factor analysis approach assists to describe general food consumption patterns that can be studied in relation to the other indicator relevant for nutritional status improvement. The aim of this study was to describe the food consumption patterns in the rural community of Kilosa District, Tanzania, and to relate it with the prevalence of food insecurity.

## Materials and Methods

### Study location

Kilosa district lies between latitude 6^0^ south and 8^0^ south and longitudes 36^0^ 30’East and 38° West. The district has a total population of 438 175 persons [5]. The district is endowed with an abundant agricultural land suitable for crop production which is a major economic activity for almost 84.2% of the total labour force [6]. Of recent, the area has been awash with persistent land conflicts especially between pastoralists and crop farmers who are fighting for the same piece of land. The main sources of water are piped water (22.7%), protected wells (26.9%) and 29.4% use surface water [6].

### Study design

Cross sectional study design was used. Repeated measurements were performed whereby same households were followed up in two rounds of the surveys across agricultural seasons. The first survey was conducted during the long rainy season (February–March) and a second survey post-harvest (September–October) in 2011.

### Sample size and Study population

The data was collected among 307 rural household members’ adults of age above nineteen years in Kilosa District–Morogoro Region, Tanzania.

### Methods

Dietary intake data were based on the 24-h dietary recall method and were obtained from a 307 households in rural populations of Kilosa District. A mother or a person responsible for meal preparation was requested to provide information on all the types of foods consumed by the household in the previous 24 hours prior to the survey date including food items consumed outside home. Food items were grouped into ten food groups as follows: cereals, oil and fats, pulses, legumes and nuts, vegetables, roots and tubers, fruits, fish and other sea foods, meat, milk and milk products and eggs [7]. A household was considered to have consumed a particular food group if consumed at least one food item belonging to that particular food group independent of its frequency in the previous twenty four hours preceding the survey date. Household food insecurity was assessed using the 9 item Household Food Insecurity Access Scale (HFIAS). A person responsible for meal preparation was interviewed to provide information on the modifications a household made in the diet or food consumption patterns due to limited resources to acquire food [8]. HFIAS was used to assess whether households experienced problems in accessing food during the reference period of 30 days prior to the survey date. Dietary patterns were assessed using the factor analysis method.

### Statistical analysis

The Statistical Product and Service Solution (SPSS) software version 17 was used to analyse the collected data [9]. Dietary patterns were extracted by factor analysis method. The components were extracted after varimax (orthogonal) rotation. Varimax method attempts to minimize the number of indicators that have high loading on one factor. Foods with factor loading score greater than 0.3 on a given dietary pattern indicated high intake of those foods that constituted a particular dietary pattern based on the output of the analysis. Factor analysis (varimax method) was used to derive the dietary patterns. A regression factor loading score was considered significant if it has a factor loading score greater or equal to 0.3 [10]. Two categories of dietary pattern were created as either low factor loading (<0.3) or higher factor loading (≥0.3). Food insecurity score was computed by summation of the scores given on the experience to food insecurity as reported by the household members. Two categories of food insecurity were created; food secure (score 0 to 2) and food insecure household (score >2) [11]. Correlation analysis was used to establish the existing relationship between dietary patterns factor scores and household food insecurity. Risk and odds for food insecurity as reflected by following a particular dietary pattern were determined by simple descriptive (crosstabs) analysis.

### Ethical considerations

Postgraduate research committee of the faculty of Agriculture of the Sokoine University of Agriculture reviewed the protocol and approved the study. The national institute for Medical Research (NIMR) further reviewed the protocol and issued a certificate to conduct the study. The reference for the certificate issued by NIMR is NIMR/ HQ/R.8a/VoI.IX/1I 89. The permission to conduct in Kilosa district was granted by District authority after submission of a formal letter. Verbal consent to participate in the study was obtained from the participating heads of the sampled households. Identified heads of the households were invited to a village office through their village leaders. The purpose of the study was told to the participating households and was further informed that participation to the study was voluntary and confidential. Majority of the household heads of the surveyed households did not know how to read and write hence the consent forms were read to them and they all agreed to participate in the study.

## Results

### Household adult composition

Females represented 58.4% and males 41.6% of the study household composition (Table 1). The mean age of male adults was 45.8±14.4 and of female adults was 39.3±13.9. Eighty four percent were married and 8.2% never married. Others were separated, widowed or widowers. Sixty percent did not have formal education. Crop cultivation (99%) and livestock keeping (63%) were two main occupations for inhabitants of the surveyed households. The proportion of government employees was 2.3%.

**Table 1:** Background characteristics of the 307 subjects studied, adult rural household members from the Kilosa District, Tanzania.

Variable	n	%
**Sex**		
Male	249	41.6
Female	349	58.4
Total	598	100.0
**Education level**		
Never been to school	359	60.0
Primary school education	214	35.8
Secondary school education/college	25	4.2
**Total**	**598**	**100.0**
**Marital status**		
Married	260	84.7
Never married	19	6.2
Widow	11	3.6
Widower	8	2.6
Divorce	5	1.6
Separated	4	1.3
**Total**	**307**	**100.0**
**Main occupation**		
Crop cultivation	305	99.3
Livestock keeping	193	62.9
Casual labourer	52	16.9
Employed	7	2.3
Petty trade	79	25.7

### Prevalence of food insecurity by seasons

**Fig 1:**
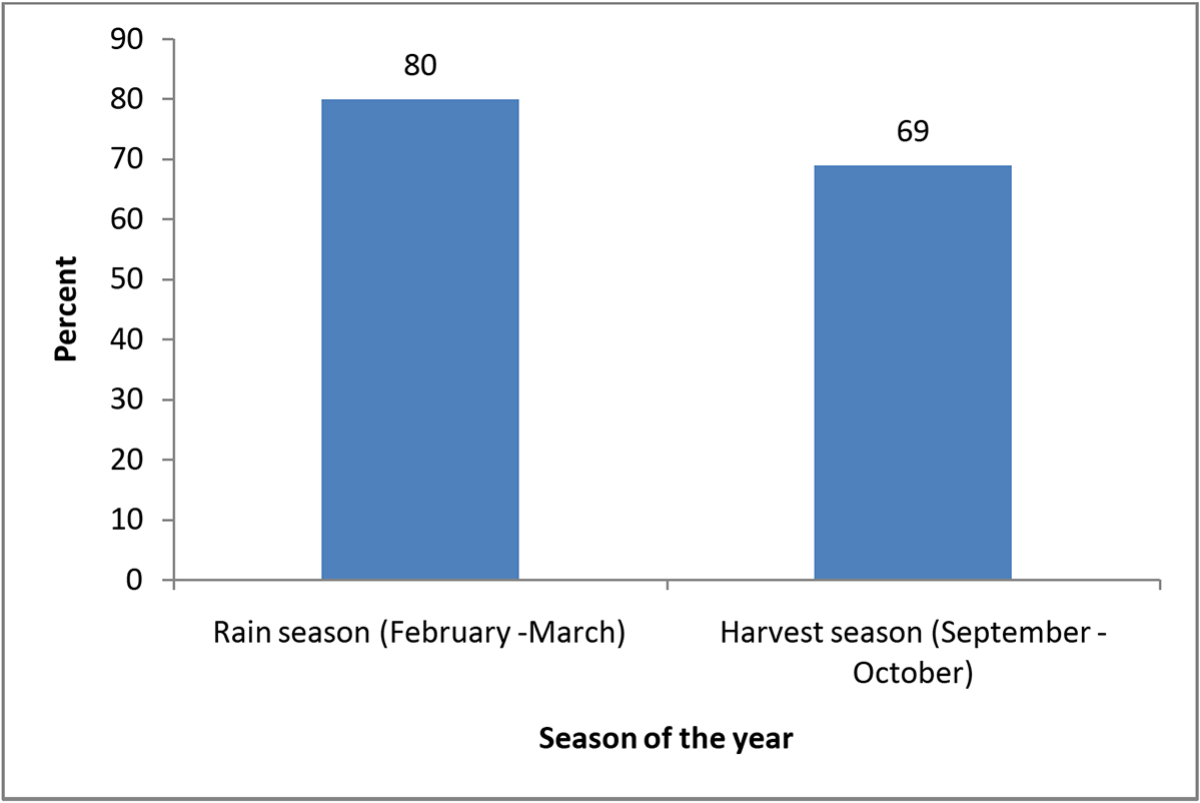
Prevalence of food insecurity by seasons among 307 subjects studied, adult rural household members from the Kilosa District, Tanzania.

About 80% and 69% of the surveyed households were food insecure during the long rainy season and immediately after harvest season, respectively (Fig 1).

### Dietary patterns

#### Harvest season

Four major dietary patterns for the population under study were identified during the harvest season (Table 2). The first pattern was meat and meat products as well as milk and milk products. This pattern was named as meat and milk consumption pattern. Other foods mostly consumed in this dietary pattern were vegetables and cooking oils. The second pattern was characterised with high consumption of pulses, legumes and nuts. Similarly households in this pattern had higher tendency to add cooking oil in their meals. Foods mostly consumed on the third dietary pattern were fish, roots and tubers. The fourth pattern was on cereals, vegetables and fruits; this was named cereals pattern. Dietary pattern I, II, III and IV explained 16.6%, 14.3%, 12.2% and 10.6% of the variability on the food consumption pattern, respectively. Factor loading of less than 0.3 was considered insignificant in the observed variation. Cumulatively, the four dietary patterns explained 53.6% of the total variance of the food consumption pattern in the surveyed population.

**Table 2:** Factor loading matrixes for the major dietary patterns during the harvest season in Kilosa, Tanzania (n = 307).

Food groups	Dietary patterns			
	I	II	III	IV
	Meat and milk /milk products	Pulses, legumes, nuts and cooking oils	Fish and other sea foods	Cereals
Cereals	0.130	0.110	0.068	-0.842
Vegetables	0.360	0.263	0.130	0.464
Roots and tubers	0.026	-0.204	-0.707	0.061
Fruits	0.034	0.404	-0.026	0.421
Meat	0.682	0.041	-0.046	-0.053
Eggs	0.628	0.107	-0.245	0.012
Fish and other sea foods	-0.084	-0.047	0.750	0.035
Pulses, legumes and nuts	-0.097	0.718	0.058	0.119
Milk and milk products	0.612	-0.443	0.275	0.112
Oils and fats	0.350	0.646	0.194	-0.113

#### Percentage of households in each dietary pattern during the harvest season

A dietary pattern characterised by higher consumption of fish (and other sea foods), roots and tubers was dominant during the harvest season (95%) (Table 3). A dietary pattern characterised with higher intake of cereals, vegetables and fruits; had relatively less number of households (58%) that consumed these foods being confirmed by relatively low factor loading of 42.3%.

**Table 3:** Percentage of households and various dietary patterns during the harvest season in Kilosa, Tanzania (n = 307).

Dietary pattern	Low factor loading		High factor loading	
	n	%	n	%
Meat and milk (DPI)	70	22.8	237	77.2
Pulses, legumes, nuts and cooking oil (DPII)	51	16.6	256	83.4
Fish (and other sea foods), roots and tubers (DPIII)	14	4.6	293	95.4
Cereals, vegetables and fruits (DPIV)	130	42.3	177	57.7

#### Percentage of food insecure households in each dietary pattern during the harvest season

Households with a dietary pattern characterised by higher consumption of meat, milk and fish as well as their products (67%) had lowest number of food insecure households (63%) compared to other dietary patterns (Table 4). On the contrary, households with lower consumption of these foods had 56% extra odds of being food insecure compared to households with higher factor loading of these foods. Households that had lower intake of cereals, vegetables and fruits had a significantly higher chance (74%) of being food insecure compared to households with higher intake of these foods.

**Table 4:** Percentage of households and various dietary patterns during the harvest season by food insecurity status in Kilosa, Tanzania (n = 307).

Dietary patterns	Dietary factor loading category	Food insecure households		Food secure households		Total		Odds* ratio	95% CI
		n	%	n	%	n	%		
Meat and milk (DPI)	Low factor loading on DPI	53	75	17	24.3	70	22.8	1.56	0.85–2.89
	High factor loading on DPI	158	66.7	79	33.3	237	77.2		
Pulses, legumes, nuts and cooking oils(DPII)	Low factor loading on DPII	37	72.5	14	27.5	51	16.6	1.25	0.64–2.23
	High factor loading on DPII	174	68	82	32	256	83.4		
Fish (and other sea foods), roots and tubers(DPIII)	Low factor loading on DPIII	10	71.4	4	28.6	14	4.6	1.14	0.35–3.74
	High factor loading DPIII	201	68.6	92	31.4	293	95.4		
Cereals, vegetables and fruits(DPIV)	Low factor loading on DPIV	98	75.4	32	24.6	130	42.3	1.74	1.05–2.87
	High factor loading on DPIV	113	63.8	64	36.2	177	57.7		

*The odds ratio of DPI or DPII or DPIII or DPIV for Low factor loading on a dietary pattern / High factor loading on a dietary pattern for food insecure households

DPI = Dietary pattern 1, DPII = Dietary pattern 2, DPIII = Dietary pattern 3 and DPIV = Dietary pattern 4.

### Risk estimate by dietary pattern and food security status

The risk for low consumption of foods such as cereals, vegetables and fruits was higher {1.18 (95% CI: 1.02–1.37)} among food insecure households compared to food secure households {0.68 (95% CI: 0.48–0.98)} (Table 5). Similarly, the risk for low consumption of foods such as meat and milk was higher {1.14 (0.97–1.33)} among food insecure households compared to food secure households {0.73 (0.46–1.14)}.

**Table 5:** Risk estimate by dietary pattern and food security status among 307 subjects studied, adult rural household members from the Kilosa District, Tanzania.

Dietary pattern	Risk estimate	
	Risk ratio* for food insecure household (95% CI)	Risk ratio^+^ for food secure household (95% CI)
Meat and milk	1.14 (0.97–1.33)	0.73 (0.46–1.14)
Pulses, legumes, nuts and cooking oils	1.07 (0.88–1.29)	0.86 (0.53–1.39)
Fish (and other sea foods), roots and tubers	1.04 (0.74–1.46)	0.91 (0.39–2.12)
Cereals, vegetables and fruits	1.18 (1.02–1.37)	0.68 (0.48–0.98)

*Risk ratio of low factor loading on a dietary pattern / high factor loading on a dietary pattern for food insecure household

+Risk ratio of low factor loading on a dietary pattern / high factor loading on a dietary pattern for food insecure household

### Correlations of dietary patterns factor loadings scores with food insecurity score

Food insecurity score was negatively related with all dietary pattern scores (Table 6).

**Table 6:** Correlates of dietary patterns factor loadings scores for harvest season in Kilosa, Tanzania (n = 307).

Variables	Household food insecurity score	REGR factor score 1 for Pattern I	REGR factor score 2 for Pattern II	REGR factor score 3 for Pattern III	REGR factor score 4 for Pattern IV
Household food insecurity score	1				
REGR factor score 1 for Pattern I	–0.32 (0.00)^**^	1			
REGR factor score 2 for Pattern II	–0.15 (0.01)^**^	0.00(1.00)	1		
REGR factor score 3 for Pattern III	–0.12(0.03)^*^	0.00(1.00)	0.00(1.00)	1	
REGR factor score 4 for Pattern IV	–0.02(0.69)	0.00(1.00)	0.00(1.00)	0.00(1.00)	1

** high significance correlation between variables

*Moderate significance correlation between variables

REGR factor score 1 (dietary pattern I) had high factor loading on Meat and milk, REGR factor score 2 (dietary pattern II) had high factor loading on Pulses, legumes and nuts, REGR factor score 3 (dietary pattern III) had high factor loading on Fish (and other sea foods), roots and tubers and REGR factor score 4 (dietary pattern IV) had high factor loading on Cereals, vegetables and fruits.

### Identified dietary patterns during the rainy season

Four major dietary patterns were extracted from the factor analysis for the population under study during the rainy season (Table 7). The first pattern was on fruits, cooking oils, fats, roots and tubers. The second pattern was named as high protein and fat dietary pattern characterized by high factor loading on eggs, meat, milk and milk products. The third dietary pattern was fish, other sea foods, vegetables, roots and tubers. The fourth pattern was characterized by high intake of pulses, legumes, nuts, cereals and vegetables. In the fourth dietary pattern, vegetables were utilised as relishes for cereals. The first, second, third and fourth dietary patterns explained 16.6%, 12.3%, 11.4% and 10.7% of food consumption variability, respectively. Cumulatively, the four dietary patterns explained 50.9% of the total variance in food consumption.

**Table 7:** Factor loading matrix for major dietary patterns during rainy season among 307 subjects studied, adult rural household members from the Kilosa District, Tanzania.

Food groups	Dietary patterns			
	I	II	III	IV
	Fruits, cooking oils, fats, roots and tubers	Eggs, meat, milk and milk products	Fish, other sea foods, vegetables, roots and tubers	Pulses, legumes, nuts, cereals and vegetables
Cereals	0.198	-0.048	-0.097	0.552
Vegetables	0.137	0.066	0.659	0.427
Roots and tubers	0.523	0.215	0.383	-0.109
Fruits	0.691	0.097	0.013	-0.024
Meat	0.056	0.661	-0.060	0.004
Eggs	0.092	0.740	-0.079	-0.031
Fish and other sea foods	0.137	0.067	-0.723	0.307
Pulses, legumes and nuts	0.473	-0.090	-0.079	-0.650
Milk and milk products	-0.017	0.530	0.180	0.031
Oils and fats	0.654	-0.049	-0.121	0.257

#### Percentage of households in each dietary pattern during the rainy season

About 80% of the surveyed households consumed diets with high consumption of pulses, legumes, nuts, cereals and vegetables (Table 8). A dietary pattern characterised with higher intake of fish and vegetables had less number of households that consumed these foods (58%).

**Table 8:** Percentage of households in each dietary pattern during the rainy season among 307 subjects studied, adult rural household members from the Kilosa District, Tanzania.

Dietary pattern	Low factor loading		High factor loading	
	n	%	n	%
Fruits, cooking oils, fats, roots and tubers (DPI)	77	25.1	230	74.9
Eggs, meat, milk and milk products (DPII)	80	26.1	227	73.9
Fish, other sea foods, vegetables, roots and tubers (DPIII)	128	41.7	179	58.3
Pulses, legumes, nuts, cereals and vegetables (DPIV)	62	20.2	245	79.8

#### Percentage of food insecure households in each dietary pattern during the rainy season

Households with a dietary pattern characterised by higher consumption of fruits, cooking oils, fats, roots and tubers had high proportion of food insecure households (82%) compared to other dietary patterns (Table 9). Households with low factor loading on this dietary pattern had 0.68 (95% CI: 0.37–1.27) odds of being food insecure compared to households with higher factor loading of the same dietary pattern.

**Table 9:** Percentage of households in each dietary pattern during rainy season by food insecurity status in Kilosa, Tanzania (n = 307).

Dietary patterns	Dietary factor loading category	Food insecure households		Food secure households		Total		Odds* ratio	95% CI
		n	%	n	%	n	%		
Fruits, cooking oils, fats, roots and tubers(DPI)	Low factor loading on DPI	58	75.3	19	24.7	77	25.1	0.68	0.37–1.26
	High factor loading on DPI	188	81.7	42	18.3	230	74.9		
Eggs, meat, milk and milk products(DPII)	Low factor loading on DPII	63	78.8	17	21.2	80	26.1	0.89	0.48–1.67
	High factor loading on DPII	183	80.6	44	19.4	227	73.9		
Fish, other sea foods, vegetables, roots and tubers(DPIII)	Low factor loading on DPIII	103	80.5	25	19.5	128	41.7	1.04	0.59–1.83
	High factor loading on DPIII	143	79.9	36	20.1	179	58.3		
Pulses, legumes, nuts, cereals and vegetables(DPIV)	Low factor loading on DPIV	49	79	13	21	62	20.2	0.92	0.46–1.83
	High factor loading on DPIV	197	80.4	48	19.6	245	79.8		

*The odds ratio of DPI or DPII or DPIII or DPIV for Low factor loading on a dietary pattern / High factor loading on a dietary pattern for food insecure households

DPI = Dietary pattern 1, DPII = Dietary pattern 2, DPIII = Dietary pattern 3 and DPIV = Dietary pattern 4.

#### Risk estimate by dietary pattern and food security status during rainy season

The risk of low consumption of fish, other sea foods, vegetables, roots and tubers was higher among food insecure households {1.01 (95% CI: 0.90–1.13)} compared to food secure households {0.97 (95% CI: 0.62–1.53)} (Table 10).

**Table 10:** Risk estimate by dietary pattern and food security status among 307 subjects studied, adult rural household members from the Kilosa District, Tanzania.

Dietary pattern	Risk estimate	
	Risk ratio* for food insecure household (95% CI)	Risk ratio^+^ for food secure household (95% CI)
Fruits, cooking oils, fats, roots and tubers (DP1)	0.92 (0.8–1.06)	1.35 (0.84–2.18)
Eggs, meat, milk and milk products (DP2)	0.98 (0.86–1.11)	1.11 (0.67–1.80)
Fish, other sea foods, vegetables, roots and tubers (DP3)	1.01 (0.90–1.13)	0.97 (0.62–1.53)
Pulses, legumes, nuts, cereals and vegetables (DP4)	0.98 (0.85–1.13)	1.07 (0.62–1.85)

*Risk ratio of low factor loading on a dietary pattern / High factor loading on a dietary pattern for food insecure household

+Risk ratio of low factor loading on a dietary pattern / High factor loading on a dietary pattern for food secure household

#### Correlations of dietary patterns factor loadings scores with scores on dietary

Household food insecurity score was negatively correlated with a dietary pattern characterized by a high intake of roots and cooking oil (r = -0.12, p = 0.04). To the contrally, household food insecurity score was positively correlated with a dietary pattern characterised with high intake of vegetables and tubers (Table 11).

**Table 11:** Correlates of the dietary patterns factor loadings scores during rainy season among 307 subjects studied, adult rural household members from the Kilosa District, Tanzania.

Variables	Household food insecurity score	REGR factor score 1 for dietary pattern I	REGR factor score 2 for dietary pattern II	REGR factor score 3 for dietary pattern III	REGR factor score 4 for dietary pattern IV
Household food insecurity score	1				
REGR factor score 1 for dietary pattern I	–0.11(0.07)	1			
REGR factor score 2 for dietary pattern II	–0.04(0.47)	0.00(1.00)	1		
REGR factor score 3 for dietary pattern III	0.12(0.04)^*^	0.00(1.00)	0.00 (1.00)	1	
REGR factor score 4 for dietary pattern IV	0.02 (0.69)	0.00(1.00)	0.00(1.00)	0.00(1.00)	1

*Moderate significance correlation between variables

REGR factor score 1 (dietary pattern I) had high factor loading on fruits and cooking oils/fats, REGR factor score 2 (dietary pattern II) had high factor loading on Eggs, meat and milk, REGR factor score 3 (dietary pattern III) had high factor loading on Fish and vegetables and tubers and REGR factor score 4 (dietary pattern IV) had high factor loading on Pulses, nuts, legumes and cereals

## Discussion

### Dietary patterns

The study has attempted to establish a link between dietary pattern and household food insecurity. Four major dietary patterns were identified and the levels of food insecurity were highest in both seasons. The analysis has allowed bringing into a broader picture the severity of food insecurity and the types of foods consumed in the respective agricultural seasons. The trends in food consumption pattern depicted in this study indicate that, the type of diets consumed by the surveyed households varied between and across agricultural seasons. Four dietary patterns were identified. These indicate the habitual foods consumption pattern in the study areas. The differences observed in the dietary patterns for the rainy and harvest seasons suggest that, an agricultural season had an influence on food availability, access, utilization and stability in the supply of these foods. The dominance of foods such as roots, tubers, fish (mostly sardines), during the harvest season and that of cereals, pulses, legumes, nuts, and vegetables during the rainy season indicates that, these foods were available to the local communities and were easily accessible in the respective seasons. Similar findings revealing dominance of cereals and vegetables was reported in Ouagadougou during the dry season [12]

A higher factor loading on cereals consumption in both seasons means that cereals were consumed by majority of the surveyed households. High consumption of cereals further suggests that, cereals were abundant in the study areas. Similarly, higher factor loading score on, pulses, legumes and nuts in both seasons suggests that, cereals were mostly consumed with pulses, legumes and nuts as relishes and were mostly abundant in all the seasons. Furthermore, cooking oils were added into diets by most of the surveyed households in both seasons during the harvest season and especially when the household had higher consumption of legumes. The present study confirms observations from the previous studies that have documented shift in the dietary pattern, the dominance of cereal based diets, addition of cooking oil in developing countries as well as inadequate fruits and vegetables consumption [10]. When cereal–based staple foods dominates and diets lack food groups such as vegetables, fruits and animal–source, may pose the risk for developing micronutrient deficiencies [13]. Similar findings on food consumption among adolescent girls have shown more than 90% of girls failed to consume the recommended amount of fruits, vegetable and dairy products but they consumed >750kcal/day of solid fat and added sugars [14].

A dietary pattern characterised by higher intake of meat and meat products as well as that of milk and milk products contributed a lot to the observed difference in terms of what the surveyed communities consumed during the harvest season. This was noted by higher discrepancy contributed by the first dietary pattern during the harvest season. Similarly, fruits, cooking oils, fats, roots and tubers consumption contributed higher discrepancy in the observed food consumption pattern during the rainy season. This indicates that, while some households were able to include these foods in their diets a good number could not manage.

The present study has affirmed earlier report documenting a low consumption of vegetables particularly during the harvest season [15]. A low consumption of fruits during the harvest season and higher consumption on vegetables during the rainy season further emphasise for a need to take the advantage of the seasonality difference in the availability to maximize the intake of these foods. From this observation it could be hypothesised that rural households were likely to have inadequate fruit intake during harvest and more intake of vegetables during rainy season. Thus, in the absence of proper local processing technology to increase shelf life and utilization of these foods; rural households are likely to miss the opportunity to tap the rich diversity of nutrients from these foods. Moreover, the rich vegetables and fruits diversity during the rainy season provide evidence in support of effort geared towards promotion of sustainable food biodiversity utilisation in promoting the need for improved household dietary diversity for good nutrition and overall health. Fruits and vegetables consumption is beneficial for health in supplying the micronutrient needed by the body and have to be consumed in adequate quantities [16].

### Correlates of the four identified dietary patterns and risk for food insecurity

The strong significant and positive correlation between household dietary diversity score with a dietary pattern characterized with high consumption of meat, milk and their products indicate that, households that can afford to include these food groups in their diets were likely to consume diversified diets. Consumption of meat has been associated with better life and therefore higher food insecurity observed in this study could be associated with limited access to food due to limited financial resources.

Households that had lower factor loading on a dietary pattern (characterised with higher factor loading score on cereals, vegetables, roots and tubers) that contributed least variation in the identified dietary patterns had elevated risk of being food insecure. This indicates that these foods play a strategic role in ensuring household food security. Further informs that, vegetables if well-cooked while preserving nutrient losses can help in reducing malnutrition.

## Conclusion

Both dietary patterns and food insecurity varied by seasons with worst scenarios most prevalent during the rainy season. The risk for inadequate dietary diversity was higher among food insecure households compared to food secure households. Effort geared at alleviating household food insecurity could contribute to consumption of a wide range of food items at the household level.
